# Deep learning-based motion correction: cardiac motion artifact and image quality improvements on chest CT

**DOI:** 10.1007/s11604-026-01986-8

**Published:** 2026-05-20

**Authors:** Yoshiyuki Ozawa, Daisuke Takenaka, Masahiko Nomura, Takahiro Ueda, Hirona Kimata, Yuya Ito, Kenji Fujii, Naruomi Akino, Takeshi Yoshikawa, Masahiro Endo, Yoshiharu Ohno

**Affiliations:** 1 Department of Diagnostic Radiology, Fujita Health University School of Medicine, Toyoake, Aichi Japan; 2 Department of Radiology, Fujita Health University Okazaki Medical Center, Okazaki, Aichi Japan; 3 Joint Research Laboratory of Advanced Medical Imaging and Artificial Intelligence, Fujita Health University School of Medicine, Toyoake, Aichi Japan; 4 Canon Medical Systems Corporation, Otawara, Tochigi, Japan; 5 Department of Radiology, Fujita Health University Bantane Hospital, Nagoya, Aichi Japan; 6 Department of Diagnostic Radiology, Shizuoka Cancer Center, Sunto-Gun, Shizuoka, Japan

**Keywords:** Lung, CT, Motion correction, Artificial intelligence

## Abstract

**Purpose:**

Since the clinical application of computed tomography (CT), cardiac and respiratory motion artifacts have caused decreased image quality and reduced detection or quantitative or qualitative evaluation of lung parenchymal or vascular abnormalities on chest CT with lung window settings in patients with pulmonary diseases. Recently, a deep learning (DL)-based motion correction algorithm (CLEAR Motion) has been developed and clinically used for chest CT. We hypothesized that CLEAR Motion can significantly reduce motion artifacts on chest CT examinations relative to conventional chest CT images reconstructed without CLEAR Motion. The purpose of this study was to determine the utility of CLEAR Motion for image quality improvement in chest CT with lung window settings in patients with various pulmonary diseases.

**Materials and methods:**

Fifty-six consecutive patients with various thoracic diseases underwent non-electrocardiogram-gated chest helical CT examination using a 320-detector row CT and underwent reconstruction using the conventional reconstruction method and CLEAR Motion. To compare the quantitative image quality, the cardio-pulmonary edge distance (CPED) and slope (CPES) were measured on each CT scan in the axial plane. Comparing cardiac motion reduction capability, overall image quality, cardiac motion artifact, and region conspicuity were visually assessed in the lung window setting on the axial, coronal, and sagittal planes. The paired t-test and Wilcoxon signed-rank test were then performed.

**Results:**

The CPEDs and CPESs of the entire lung and left lung on CT with CLEAR Motion were significantly superior to those of CT without CLEAR Motion (*p* < 0.001). The overall image quality, cardiac motion artifact, and region conspicuity on CT with CLEAR Motion were significantly higher than those without CLEAR Motion on each plane (*p* < 0.001).

**Conclusion:**

The DL-based motion correction algorithm named as ‘CLEAR Motion’ has a potential to improve image quality on chest CT with lung window setting in patients with pulmonary diseases.

## Introduction

Since the clinical application of computed tomography (CT), cardiac and respiratory motion artifacts have been the main causes of decreased image quality and reduced detection or quantitative or qualitative evaluation of lung parenchymal or vascular abnormalities on chest CT with lung window settings in patients with pulmonary diseases. Many investigators have attempted not only chest CT with cone beam CT or other CT systems but also positron emission tomography (PET) and PET fused with CT (PET/CT) in patients with thoracic malignancies and other diseases [1–27]. The concurrent presence of high temporal and high spatial resolution on state-of-the-art multidetector-row CT systems with energy-integrated or photon-counting detectors opens new possibilities during chest examination, namely an optimized morphological analysis of all thoracic organs combined with functional imaging, whenever necessary. This concept of integrated cardiothoracic imaging is particularly favorable for the evaluation of respiratory disorders owing to the complex nature of the interactions between the pulmonary and cardiovascular systems [1–27]. Previously restricted to the cardiac consequences of respiratory diseases or the pulmonary consequences of cardiac disorders, these interactions now include respiratory and cardiac comorbidities in which both organs are simultaneously involved in the same disorder, independent of the direct consequences of organ dysfunction [1–27].

Although several investigators have attempted to reduce motion artifacts by applying different methods, no one has provided commercially available motion correction methods for routine chest CT in the last several decades. Recently, the Canon Medical Systems Corporation developed and clinically established a deep learning (DL)-based motion correction algorithm (CLEAR Motion: Canon Medical Systems, Otawara, Tochigi, Japan) for chest CT examinations. We hypothesize that CLEAR Motion can significantly reduce motion artifacts on chest CT examination relative to conventional chest CT reconstructed without CLEAR Motion in routine clinical practice. The purpose of this study was to determine the utility of CLEAR Motion for image quality improvement in chest CT with lung window settings in patients with various pulmonary diseases.

## Materials and methods

This retrospective study was approved by the Institutional Review Board of Fujita Health University Hospital and was compliant with the Health Insurance Portability and Accountability Act. Written informed consent was waived. This study was technically and financially supported by the Canon Medical Systems Corporation. Moreover, this study was also partly and financiallysupported by Smoking Research Foundation. Four of the authors are employees of Canon Medical Systems (H.K., Y. I., K.F., and N.A.), but do not have control over any of the data and information submitted for publication or over which data and information were to be included in this study. This study was also financially supported by Smoking Research Foundation. All the data were controlled and statistically analyzed by Y.O., D.T., M.N., T.U., T.Y., M.E. and Y.Oh.

### Subjects

**Fig. 1:**
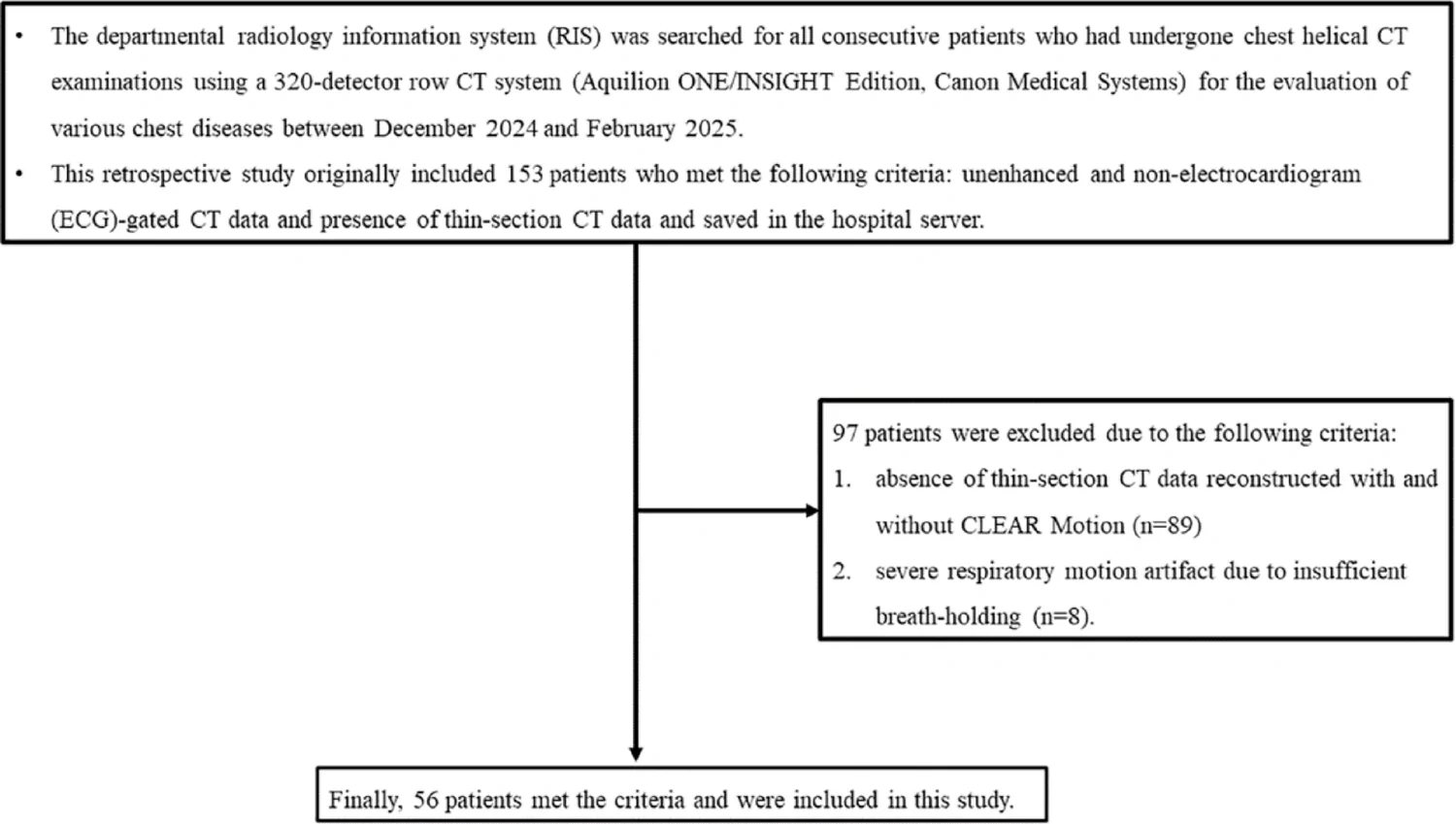
Flow diagram of the present study

The departmental radiology information system (RIS) was searched for all consecutive patients who had undergone chest helical CT examinations using a 320-detector row CT system (Aquilion ONE/INSIGHT Edition, Canon Medical Systems) for the evaluation of various chest diseases between December 2024 and February 2025. The following patient data were included in this study: (1) unenhanced and non-electrocardiogram (ECG)-gated CT data and (2) presence of thin-section CT data and saved in the hospital server. Therefore, this retrospective study included 153 patients who met the inclusion criteria. The exclusion criteria were as follows: (1) absence of thin-section CT data reconstructed with and without CLEAR Motion and (2) severe respiratory motion artifacts due to insufficient breath-holding due to coughing. From the original cohort (n = 153), 97 patients were excluded due to criteria (1) (n = 89) and criteria (2) (n = 8). Finally, 56 patients met the inclusion criteria and were included in the study. Figure 1 shows a flow diagram of this study.

### CT examination

All CT data were obtained using a 320-detector row (i.e., area-detector) CT (ADCT) scanner (Aquilion ONE/INSIGHT Edition; Canon Medical Systems). Each unenhanced CT scan of the entire lung, from the lung apex to the diaphragm, was obtained during the suspension of respiration at the end of inhalation. The details of the scan protocol are presented in Table 1. All CT data were reconstructed by DL reconstruction (Advanced. Intelligent Clear-IQ Engine: AiCE, Canon Medical Systems) with or without a DL-based motion correction algorithm (CLEAR Motion, Canon Medical Systems).

**Table 1:** Scan protocol in this study

	ADCT with CLEAR Motion	ADCT without CLEAR Motion
CT system	320-detector row CT (Aquilion ONE / INSIGHT Edition)	
Scan method	Helical scan	
Detector collimation	0.5 mm × 80rows	
Beam Pitch	0.813	
Tube voltage (kVp)	120	
Tube current (mA)	auto mA (image SD = 13)	
Rotation Time (s/rot)	0.5	
FOV (mm)	240–400	
Slice thickness (mm)	1	
Reconstruction method	Deep Learning Reconstruction (Advanced. intelligent Clear-IQ Engine: AiCE)	
Reconstruction matrix	512	
Deep learning-based motion correction algorithm (CLEAR Motion) application	with CLEAR Motion	without CLEAR Motion
Radiation dose (CTDIvol: mGy)	5.1 ± 3.1	

### Deep learning-based motion correction algorithm (CLEAR motion)

**Fig. 2:**
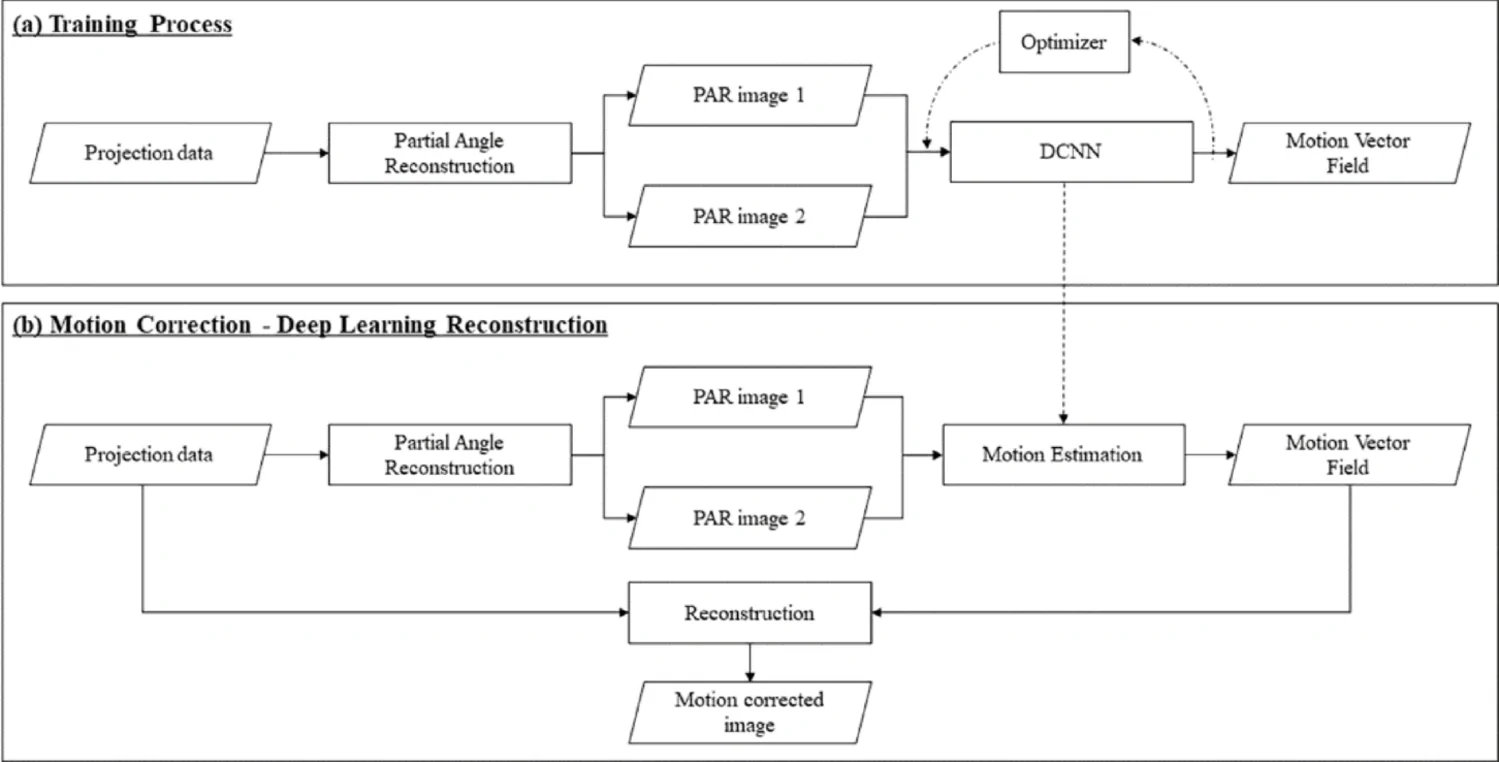
Flow chart for the DL-based motion correction algorithm (CLEAR Motion). In the training process (**a**), the full projection data are decomposed into two partial angle reconstructions (PAR). Next, multiple PAR images with different time phases are input into the DCNN, which estimates the motion vector field between the PAR images. In the motion correction process based on deep learning reconstruction (**b**), PAR images created from actual projection data are applied to the pretrained DCNN to estimate the motion vector field. Finally, a motion-corrected image is reconstructed by incorporating the DCNN-predicted motion vector field

A new motion correction method, CLEAR Motion, is a deep-learning reconstruction method that reduces motion artifacts caused by a patient’s body movement in the lung region (Fig. 2). This method consists of four steps:

CLEAR Motion estimates the amount of motion using multiple partial angle reconstruction (PAR), which uses a smaller angle than the normal reconstruction. The temporal resolution of PAR is higher than that of the normal reconstruction, allowing for a more accurate estimation of the amount of motion required for faster movements.

In the first step of the training process, multiple PAR images of different time phases created from the projection data were used as input data. It is important that the training for this method does not require motion-free reference images. This method utilizes PAR, which is generated from pairs of opposing projection data passing through the same path. If there are no temporal changes in the object, PAR image 1 and PAR image 2 will match each other. In contrast, differences between a pair of PAR images represent the vector field caused by patient motion, described as the motion vector field (MVF). The MVF to be estimated is the vector field that warps one PAR image to match the other PAR image. This is calculated by general non-rigid image registration. However, high-accuracy non-rigid registration is computationally expensive. Therefore, in CLEAR Motion, PAR images are used as input data, and the network is pretrained using the ideal MVFs obtained by non-rigid image registration as the target (Fig. 2a). For network training, a large-scale dataset of clinical data acquired in the lung region was used. The dataset was extensively collected to cover all scan conditions supported by this function, including variations in helical pitch and gantry rotation speed.

In the second step, the amount of movement (i.e., MVF) is estimated from PAR images, and a deep convolutional neural network (DCNN) is pretrained to obtain the amount of motion from multiple PAR images. Using a large amount of clinical data, a DCNN, which enables a highly accurate estimation of various motions, is built. In the DL reconstruction process, multiple PAR images are created from the acquired projection data, and these PAR images are input into the DCNN to estimate the amount of motion from the acquired data. In Motion Correction–Deep Learning Reconstruction (MC-DLR), multiple PAR images are generated from the acquired projection data separately from the standard reconstruction process. Pairs of these PAR images are then input into a pre-trained DCNN to estimate the motion. The estimated motion is subsequently used to correct the projection paths during the back-projection step of the conventional image reconstruction process (Fig. 2b). The MC-DLR reconstruction process does not require iterative optimization; instead, motion estimation and correction are achieved by applying the trained DCNN only once, enabling fast motion estimation and correction. Moreover, MC-DLR can be combined with DLR (AiCE) and super-resolution DLR (Precise IQ Engine: PIQE, Canon Medical Systems), allowing the generation of high-quality images with reduced motion artifacts, decreased image noise, and improved spatial resolution.

In the last step, using the estimated motion, reconstruction is performed while modifying the projection path of the back projection in the normal image reconstruction. CLEAR Motion reconstruction does not require iterative processing and only requires the application of the pretrained DCNN once, achieving high-speed reconstruction that is applicable to everyday clinical practice.

### Image analysis

All CT images were randomized for independent review by two board-certified chest radiologists (Y. Oh. and D.T.), with 31 and 34 years of experience, respectively. For this study, all CT data sets in the lung window setting (level: − 550 HU, width: 1600 HU) were randomly interpreted using a picture archiving and communication system (RapideyeCore TFS01; Canon Medical Systems) with the reviewers having no access to information about the technical parameters. All CT image evaluations were quantitatively and qualitatively performed by two radiologists at different times and days, and in different reading rooms.

#### Quantitative image quality analysis

To compare quantitative image quality on CT images with and without CLEAR Motion, images at the aortic arch, carina, inferior pulmonary vein, and lung basis levels were used for a quantitative analysis. First, circular regions of interest (ROIs) of 10 mm in diameter were placed on the lung window setting at the tracheal lumen and bilateral lung parenchyma on CT data reconstructed without CLEAR Motion, and all ROIs were copied to CT data reconstructed with CLEAR Motion. Lung parenchymal CT values and image noise within both lungs were determined for each patient as the mean CT value and standard deviation (SD) within the ROI in both lungs at different levels [28–34]. Image noise within the trachea was also determined as the SD of the CT value within the ROI of the trachea [28–34]. Therefore, a total of 896 ROI measurements were performed (2 reconstruction methods × 8 ROIs × 56 patients) to determine all indices as well as the signal-to-noise ratio (SNR). The SNR was determined using the following formula (28–34):

1 $$\begin{gathered} {\mathrm{SNR}}_{{{\mathrm{Lung}}}} = \left| {{\mathrm{mean}}\,{\mathrm{CT}}\,{\mathrm{value}}\,{\mathrm{within}}\,{\mathrm{the}}\,} \right. \hfill \\ \quad \quad \quad \quad \left. {{\mathrm{ROI}}\,{\mathrm{in}}\,{\mathrm{lung}}\,{\mathrm{parenchyma}}} \right|/{\mathrm{SD}}\,{\mathrm{within}}\, \hfill \\ \quad \quad \quad \quad {\mathrm{the}}\,{\mathrm{ROI}}\,{\mathrm{in}}\,{\mathrm{the}}\,{\mathrm{trachea}} \hfill \\ \end{gathered}$$

where SD is the standard deviation within the trachea as the background.

**Fig. 3:**
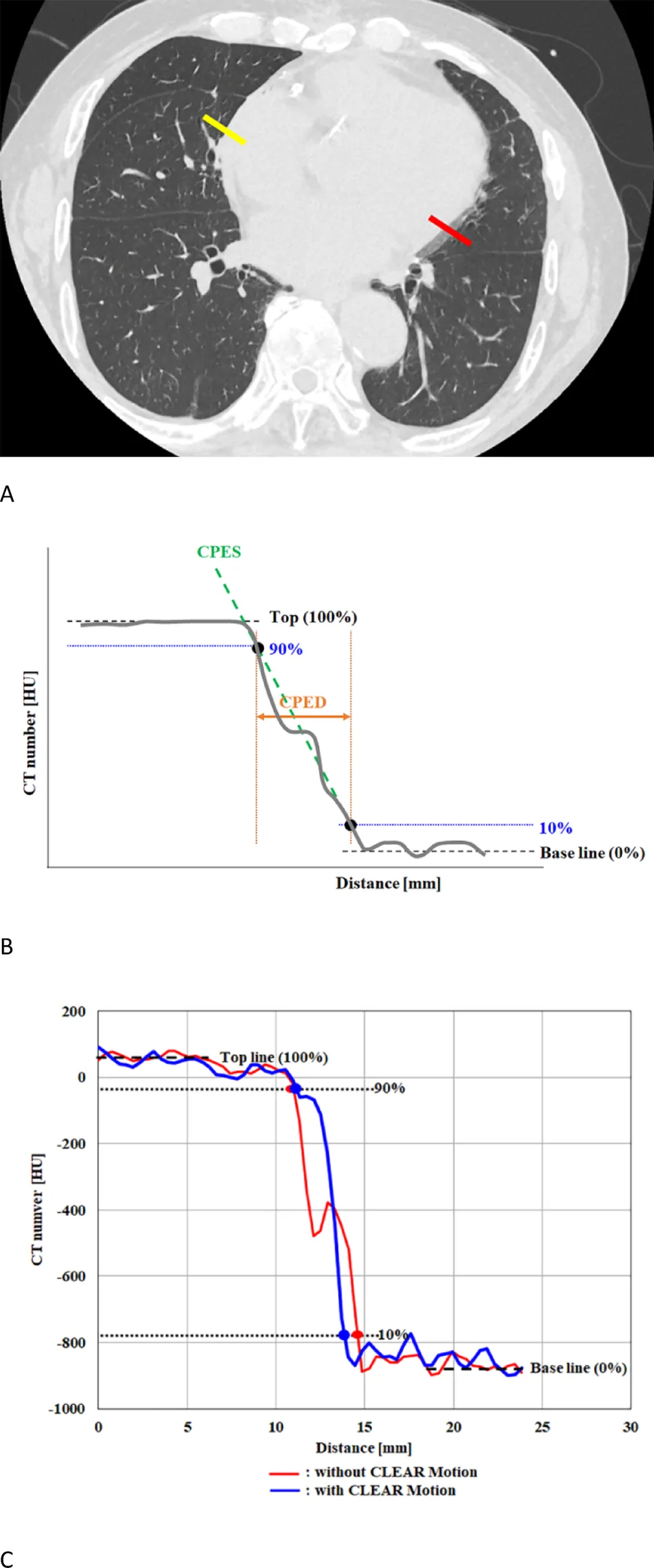
An example of the cardio-pulmonary borders in the right and left lungs (yellow line and red line) and schematic illustration of evaluation. **A**: On the CT image at the inferior pulmonary vein level, cardiopulmonary borders in the right (yellow line) and left (red line) lungs were manually evaluated to measure cardio-pulmonary edge distance (CPED) and slope (CPES) from the profile curves of each line. **B**: In each subject, the CPED (labeled orange line) and CPES (labeled green line) at a pixel attenuation from 10 to 90% of the maximum CT attenuation are shown. The CPED measured 10% and 90% of the x-axis distance. The CPES measured the slope of the line connecting the 10% and 90% points from the baseline. **C**: Examples of profile curves for CT images with and without CLEAR Motion at the inferior pulmonary vein level, cardiopulmonary borders in the left lungs (red line in Fig. 3A) are demonstrated

Moreover, cardio-pulmonary edge distance (CPED) and slope (CPES) in each patient were also measured from the profile curve of the right and left cardiopulmonary borders, whose length was almost 25 mm (0.39 mm/pixel × 64 pixel = 24.96 mm), at the inferior pulmonary vein level (2 reconstruction methods × 1 level × 2 profile curves × 56 patients = 224 curves) for each patient (Fig. 3). Moreover, each line was drowned as the perpendicular to heart–lung boarder at the position, whose fat layer was the smallest between the myocardium and the lung.

According to previous literatures [33, 35], Image J software and its particle analysis tool (Plot Profile) were used to generate the profile curves in this study. To assess the capability for improvement of cardiac motion artifact with CLEAR Motion, profile curve at each cardiopulmonary border, measured by the 10–90% CPED and CPES were measured. To calculate each index, base lines at top and basal level of profile curve were determined as the average CT values from 15 pixels (0.39 mm/pixel × 15 pixel = 5.85 mm) from the start and end points of line in all patients. Then, CPRS was calculated by the following formula:

2 $${\mathrm{CPES}} = \left( {{\mathrm{CT}}_{{{9}0\% }} - {\mathrm{CT}}_{{{1}0\% }} } \right)/{\mathrm{CPED}}$$

CT_90%_ was determined as pixel attenuation from 90% of the maximum CT attenuation and CT_10%_ was evaluated as pixel attenuation from 10% of the maximum CT attenuation.

All quantitative index evaluations were performed in the axial plane by the same two board-certified chest radiologists, and the final values were determined as the average value for each patient.

#### Qualitative assessment of image quality

To compare the cardiac motion reduction capability of CLEAR Motion, the same two board-certified chest radiologists evaluated the overall image quality using a 5-point visual scoring system as follows: 1, non-diagnostic; 2, poor; 3, acceptable; 4, good; and 5, excellent. Moreover, cardiac motion artifacts were assessed using a 5-point visual scoring system as follows: 1, absent; 2, mild; 3, moderate; 4, severe; and 5, non-diagnostic. In addition, artifacts other than cardiac motion, which were determined as the sum of noise, streak artifact, and blurring of the border between the lung and chest wall, were also assessed using a 5-point visual scoring system as follows: 1, absent; 2, probably absent; 3, equivocal; 4, probably present; and 5, present.

To determine the clinical utility of CLEAR Motion for lesion depiction within lung zones close to cardio-pulmonary borders in both lungs, lesion conspicuity was determined as follows: 1, impossible to evaluate due to cardiac motion artifact or other artifacts; 2, depicted, but difficult to evaluate due to cardiac motion artifact; 3, depicted to other radiological findings or fake radiological findings due to cardiac motion artifact; 4, insufficiently depicted as original radiological findings due to cardiac motion artifact; and 5, depicted without less cardiac motion artifact. The final value for each qualitative image quality index score was determined as consensus of two readers in all CT data.

These systems were used to evaluate the aortic arch, carina, inferior pulmonary vein, and lung basis levels on the axial, coronal, and sagittal planes on each CT image.

### Statistical analysis

For quantitative image quality comparisons, CT values, SNR, CPED, and CPES of the entire, right, and left lungs were compared between CT data with and without CLEAR Motion using paired t-tests.

To determine the interobserver agreement for qualitative image quality index assessment on each CT, kappa statistics followed by the χ^2^ test were performed [36]. Agreement was considered poor for κ < 0.21, fair for κ = 0.21–0.40, moderate for κ = 0.41–0.60, substantial for κ = 0.61–0.80, and excellent for κ = 0.81–1.00 [36]. The overall image quality, cardiac motion artifacts, and other artifacts other than cardiac motion on each plane were compared between CT data with and without CLEAR motion using Wilcoxon’s signed rank test.

Interobserver agreements for the six major radiological findings on each CT were evaluated using kappa statistics, followed by the χ^2^ test [36]. Agreement was considered poor for κ < 0.21, fair for κ = 0.21–0.40, moderate for κ = 0.41–0.60, substantial for κ = 0.61–0.80, and excellent for κ = 0.81–1.00 [36]. A receiver operating characteristic (ROC) analysis was performed to compare the diagnostic performance of each radiological finding between CT data with and without CLEAR Motion.

*P* values of < 0.05 were considered to indicate statistical significance in all statistical analyses. All statistical analyses were performed using JMP (ver. 14: SAS Institute Japan, Tokyo, Japan; StatMate III) or EZR (ver. 1.54: https://www.jichi.ac.jp/saitama-sct/SaitamaHP.files/statmedEN.html) [37].

## Results

**Fig. 4:**
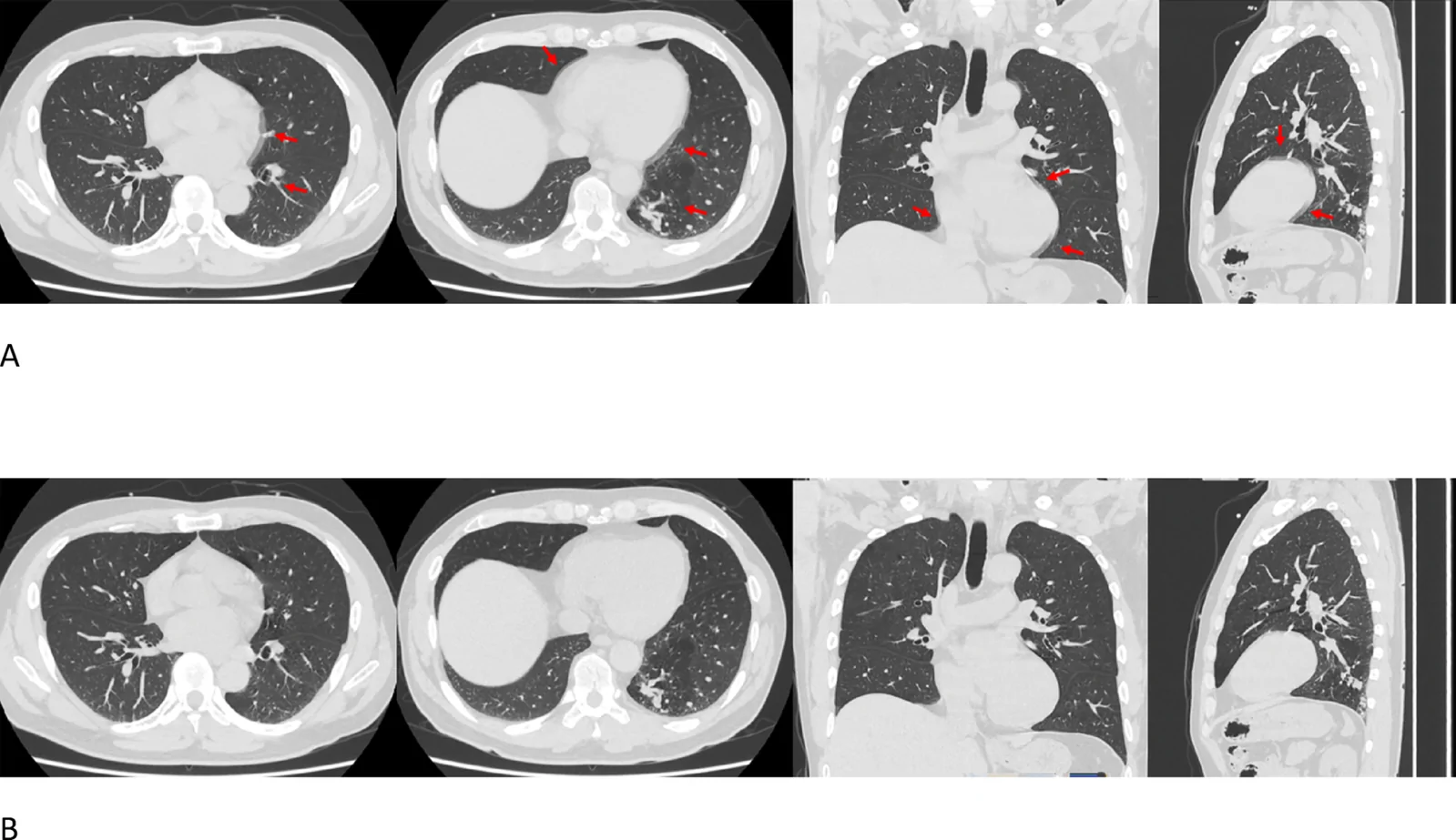
A 42-year-old male with pulmonary sequestration. **A**: (L to R: axial CT (cranial to caudal), coronal multiplanar reformatted (MPR), and sagittal MPR images from CT data without CLEAR Motion). Axial CT images and coronal and sagittal MPR images show cardiac motion artifacts (red arrows) at the cardio-pulmonary borders in both lungs. **B**: (L to R: axial CT (cranial to caudal), coronal multiplanar reformatted (MPR), and sagittal MPR images from CT data without CLEAR Motion). All cardiac motion artifacts clearly disappeared, and the borders between the sequestrated lung and normal lung, pulmonary vasculatures, and bronchi were clearly depicted

Representative case is shown in Fig. 4. The success rate of motion correction was 100% in this study.

Table 2 shows details of patient characteristics. This study group of 56 patients (mean age, 68 years; age range, 30–86 years) included 38 males (mean age, 66 years; age range, 50–86 years) and 18 females (mean age, 72 years; age range, 30–82 years) with interstitial lung abnormalities (n = 18), interstitial lung diseases (n = 16), lung cancers (n = 11), pulmonary emphysema (n = 6), nontuberculous mycobacteria (NTM) infections (n = 4) and pulmonary sequestration (n = 1).

**Table 2:** Patient characteristics

Sex (cases)	Male		38
	Female		18
Age (years)	Mean ± SD		68 ± 11
	(range)		(30–86)
Underlying disease (cases)	Interstitial lung abnormality		18
	Interstitial lung disease		16
		Systemic Sclerosis (SSc)	(6)
		Polymyositis/dermatomyositis (PM/DM)	(5)
		Rheumatoid arthritis (RA)	(3)
		Mixed connective tissue disorder (MCTD)	(2)
	Lung cancer		11
		Invasive adenocarcinoma	(5)
		Adenocarcinoma with other subtypes	(3)
		Squamous cell carcinoma	(2)
		Minimally invasive adenocarcinoma	(1)
	Pulmonary emphysema		6
	Nontuberculous mycobacteria (NTM)		4
	Pulmonary sequestration		1

The results of the quantitative image quality indices are listed in Table 3. On comparing quantitative image quality indices, the image noises within the entire right lung and left lung on CT with CLEAR Motion were significantly higher than those on CT without CLEAR Motion (entire lung: *p* < 0.001, right lung: *p* < 0.001, left lung: *p* < 0.001). Moreover, the SNRs within the entire right lung and left lung on CT with CLEAR Motion were significantly lower than those on CT without CLEAR Motion (entire lung: *p* < 0.001, right lung: *p* < 0.001, left lung: *p* < 0.001). In addition, CPEDs of the entire lung and left lung on CT with CLEAR Motion were significantly shorter than those of CT without CLEAR Motion (entire lung: p = 0.001, left lung: *p* < 0.001). Furthermore, CPESs of the entire lung and left lung on CT with CLEAR Motion were significantly larger than those of CT without CLEAR Motion (entire lung: p = 0.001, left lung: *p* < 0.001).

**Table 3:** Comparisons of quantitative image quality indices between CT data with and without CLEAR Motion

			CT with CLEAR Motion	CT without CLEAR Motion	p value
CT value	Whole lung	(mean ± SD)	− 882.1 ± 68.6	− 885.4 ± 35.4	0.87
	Right lung	(mean ± SD)	− 887.1 ± 38.9	− 887.3 ± 39.0	0.79
	Left lung	(mean ± SD)	− 881.2 ± 89.1	− 883.6 ± 31.4	0.87
Image noise	Whole lung	(mean ± SD)	43.0 ± 17.5	34.9 ± 18.7	< 0.0001
	Right lung	(mean ± SD)	40.9 ± 14.5	33.5 ± 15.7	< 0.0001
	Left lung	(mean ± SD)	45.1 ± 19.8	36.4 ± 21.2	< 0.0001
SNR	Whole lung	(mean ± SD)	41.5 ± 6.2	64.6 ± 7.2	< 0.0001
	Right lung	(mean ± SD)	41.8 ± 5.8	64.8 ± 7.4	< 0.0001
	Left lung	(mean ± SD)	41.3 ± 6.7	64.4 ± 7.1	< 0.0001
CPED	Whole lung	(mean ± SD)	2.0 ± 1.6	2.8 ± 2.7	0.001
	Right lung	(mean ± SD)	2.1 ± 1.5	2.0 ± 1.4	0.63
	Left lung	(mean ± SD)	2.0 ± 1.6	3.6 ± 3.4	0.0001
CPES	Whole lung	(mean ± SD)	486.3 ± 233.3	430.1 ± 252.1	0.001
	Right lung	(mean ± SD)	467.8 ± 213.3	497.2 ± 238.0	0.80
	Left lung	(mean ± SD)	504.9 ± 252.3	361.7 ± 249.7	<0.001

SNR: signal-to-noise ratio, CPED: cardio-pulmonary edge distance, CPES: cardio-pulmonary edge slope, SD: standard deviation

Table 4 shows the interobserver agreement of each quantitative image quality index on CTs with and without CLEAR Motion on the axial, coronal and sagittal planes. Interobserver agreement for all qualitative image quality indices was determined to be significant and almost perfect (κ = 0.81 to ≤ 0.99, *p* < 0.001).

**Table 4:** Interobserver agreement of each quantitative image quality index on CT with and without CLEAR Motion on the axial, coronal and sagittal planes

		Axial plane		Coronal plane		Sagittal plane	
		CT with CLEAR Motion	CT without CLEAR Motion	CT with CLEAR Motion	CT without CLEAR Motion	CT with CLEAR Motion	CT without CLEAR Motion
Overall image quality	κ value	0.83	0.9	0.94	0.88	0.89	0.82
	*p* value	< 0.001	< 0.001	< 0.001	< 0.001	< 0.001	< 0.001
Cardiac motion artifact	κ value	0.84	0.87	0.85	0.85	0.89	0.84
	*p* value	< 0.001	< 0.001	< 0.001	< 0.001	< 0.001	< 0.001
Other artifacts except cardiac motion artifact	κ value	0.88	0.81	0.88	0.87	0.82	0.84
	*p* value	< 0.001	< 0.001	< 0.001	< 0.001	< 0.001	< 0.001
Lesion depiction	κ value	0.89	0.9	0.92	0.96	0.96	0.99
	*p* value	< 0.001	< 0.001	< 0.001	< 0.001	< 0.001	< 0.001

The results of the qualitative image-quality indices are compared in Table 5. On comparison of overall image quality on the three planes, all overall image qualities on CT with CLEAR Motion were significantly higher than that on CT without CLEAR Motion (axial plane: *p* < 0.001, coronal plane: *p* < 0.001, sagittal plane: median, *p* < 0.001). Moreover, when comparing cardiac motion artifacts on each plane, cardiac motion artifacts on CT with CLEAR Motion were significantly lower than those on CT without CLEAR Motion (axial plane, *p* < 0.001; coronal plane, *p* < 0.001; sagittal plane, *p* < 0.001). In addition, artifacts other than cardiac motion artifacts on the axial plane on CT with CLEAR Motion was significantly higher than that on CT without CLEAR Motion (*p* < 0.001). Furthermore, the lesion depiction of CT with CLEAR Motion was significantly higher than that without CLEAR Motion on each plane (*p* < 0.001).

**Table 5:** Comparison of qualitative image quality indices between CT data with and without CLEAR Motion

		Axial plane		Coronal plane		Sagittal plane	
		CT with CLEAR Motion	CT without CLEAR Motion	CT with CLEAR Motion	CT without CLEAR Motion	CT with CLEAR Motion	CT without CLEAR Motion
Overall image quality	Median	5	4	5	5	5	5
	IQR	5–5	4–5	4–5	4–5	4–5	4–5
	*p* value	< 0.001		< 0.001		< 0.001	
Cardiac motion artifact	Median	1	2	1	1	1	1
	IQR	1–1	1–2	1–1	1–2	1–1	1–2
	*p* value	< 0.001		< 0.001		< 0.001	
Artifacts other than cardiac motion artifacts	Median	2	2	1	1	2	2
	IQR	2–2	1–2	1–2	1–2	2–3	2–3
	*p* value	< 0.001		0.277		0.0858	
Lesion depiction	Median	5	4	5	5	5	5
	IQR	5–5	4–5	4–5	4–5	4–5	4–5
	*p* value	< 0.001		< 0.001		< 0.001	

IQR, interquartile range

## Discussion

Our study results indicate that a newly developed and commercially available DL-based motion correction algorithm (CLEAR Motion) could reduce cardiac motion artifacts and improve image quality on thin-section CT on all planes without ECG-gating in patients with pulmonary diseases. To the best of our knowledge, this study is the first to test DL-based motion correction algorithm and compare the capability of CT with CLEAR Motion for quantitative and qualitative image quality and region conspicuity with that without CLEAR Motion in patients with pulmonary diseases. Moreover, this technique would be better to be applied for more accurate evaluations on quantitative assessment of lung parenchyma change for interstitial lung disease or others, airway dimension evaluation in chronic obstructive pulmonary disease or computer-aided volumetry for pulmonary nodule on CT with CLEAR Motion as compared with that without CLEAR Motion.

The comparison of quantitative image quality indices revealed that cardiac motion artifacts assessed as whole- and left-lung CPEDs and CPESs were significantly improved. These facts demonstrate that CLEAR Motion could reduce cardiac motion artifacts, especially at the left cardio-pulmonary borders. On the other hand, image noise within the entire, right, and left lungs of CT with CLEAR Motion were significantly higher than those of CT without CLEAR Motion. Moreover, SNRs within the entire, right, and left lungs of CT with CLEAR Motion were significantly reduced relative to CT without CLEAR Motion, although CT values within the lung showed no significant differences between CT with and without CLEAR Motion. In CLEAR Motion, teaching CT data is generated from the sinogram of the original CT data subdivided into smaller sections in time and representing these data in the domain as partial angle reconstructions (PARs). DCNN was trained to generate a motion vector field from the original CT and generated CT data with PARs. Then, CLEAR Motion estimates the motion vector field using this DCNN and places it in the back projection process for CT reconstruction and the reconstructed CT has fewer cardiac motion artifact images. Therefore, CLEAR Motion prioritizes temporal resolution relative to normal reconstruction, resulting in fewer motion artifacts but more image noise. Considering the abovementioned process, our results may be expected and considered compatible with the basics of CLEAR Motion in routine clinical practice. Although the success rate of CLEAR Motion in this study was 100%, mean image noise or SNR of CT with CLEAR Motion were closed to or reduced-dose CT on conventional area-detector or high-definition CTs reconstructed with filter back projection or hybrid-type iterative reconstruction methods [28, 34]. When considering the past study results [28, 34], CT image obtained with CLEAR Motion in this study was considered as clinically applicable. Therefore, further improvement of CLEAR Motion for decreasing image noise and increasing SNR are considered as requested for future clinical application, if needed. Moreover, these facts might also be suggested that CT obtained without CLEAR Motion can be reduced radiation dose in routine clinical practice.

When assessing the interobserver agreement of each quantitative image quality index on CTs with and without CLEAR Motion on all planes, interobserver agreement was determined to be significant and almost perfect on all planes. Therefore, our qualitative evaluations were considered reproducible according to the relevant literature [36].

The comparison of qualitative image quality and lesion depiction assessments revealed that overall image quality, cardiac motion artifacts, and lesion depiction were significantly improved by CLEAR Motion on all three planes, while other artifacts except cardiac motion artifacts significantly remain on CT with CLEAR Motion on the axial plane. In this study, artifacts other than cardiac motion artifacts were determined as the sum of noise, streak artifacts, and blurring of the border between the lung and chest wall. According to our study results, these artifacts were more prominent on the axial plane and less visible on the coronal and sagittal planes. Therefore, further improvements in CLEAR Motion are required to improve the image quality in routine clinical practice.

The present study was associated with several limitations. First, the study cohort was limited, and a larger prospective cohort study is required. Moreover, the investigators in this study were only two radiologists, whose experiences had high experience and it would be better to include equal to or more than three radiologists including slightly less experienced radiologists for reducing the bias and improving the robustness of the evaluation. Second, the CT data with and without CLEAR Motion were provided by a single vendor and were not compared with those provided by other vendors. Moreover, we did not test other reconstruction methods, such as hybrid-type and model-based iterative reconstructions, as well as other CT systems, including photon-counting detector CT in this study. These factors would have affected our study results. Third, we did not directly compare diagnostic performance, quantitative morphological evaluation capability for pulmonary functional CT imaging, or its effect on decision-making in relation to patient treatment. According to above-mentioned our study results, CLEAR motion would be better to be retrospectively applied to reduce cardiac motion artifact for qualitative and quantitative evaluations of lung texture, airway dimension, mucus plaque or intra-pulmonary arterial defect on chest CT data, which is determined as having cardiac motion artifact and considered to have influence to each evaluation result based on clinical aims in patients with different pulmonary diseases. Therefore, CT images with CLEAR motion may be interpreted as alone rather than together with conventional CT images in routine clinical practice. However, the clinical relevance of CLEAR motion for patient management or care has not been determined in this study, and further studies using a large prospective cohort with ontological diseases are warranted. Fourth, this study did not include an in vitro study, and detailed analyses for spatial resolution improvements were not performed, and further investigations with phantoms are warranted.

In conclusion, a DL-based motion correction algorithm named as CLEAR Motion has the potential to improve image quality on chest CT with lung window settings in patients with pulmonary diseases.

## Abbreviations

ADCT: Area-detector CT
CE: Contrast-enhanced
CPED: Cardio-pulmonary edge distance
CPES: Cardio-pulmonary edge distance slope
ECG: Electrocardiogram
HDCT: High-definition CT
SD: Standard deviation
SNR: Signal-to-noise ratio
