# Correction to: Deep learning-based motion correction: cardiac motion artifact and image quality improvements on chest CT

**DOI:** 10.1007/s11604-026-02018-1

**Published:** 2026-07-09

**Authors:** Yoshiyuki Ozawa, Daisuke Takenaka, Masahiko Nomura, Takahiro Ueda, Hirona Kimata, Yuya Ito, Kenji Fujii, Naruomi Akino, Takeshi Yoshikawa, Masahiro Endo, Yoshiharu Ohno

**Affiliations:** 1 Department of Diagnostic Radiology, Fujita Health University School of Medicine, Toyoake, Aichi Japan; 2 Department of Radiology, Fujita Health University Okazaki Medical Center, Okazaki, Aichi Japan; 3 Joint Research Laboratory of Advanced Medical Imagingand Artificial Intelligence, Fujita Health University School of Medicine, Toyoake, Aichi Japan; 4 Canon Medical Systems Corporation, Otawara, Tochigi Japan; 5 Department of Radiology, Fujita Health University Bantane Hospital, Nagoya, Aichi Japan; 6 Department of Diagnostic Radiology, Shizuoka Cancer Center, Shunto-Gun, Shizuoka, Japan

**Correction to** **:** **Japanese Journal of Radiology**

https://doi.org/10.1007/s11604-026-01986-8

In Table 3 of the original publication the entry “CPES” and its corresponding values were inadvertently omitted.

The incorrect and the corrected versions of Table 3 are given below.

Incorrect version:

**Table 3:** Comparisons of quantitative image quality indices between CT data with and without CLEAR Motion

			CT with CLEAR Motion	CT without CLEAR Motion	p value
**CT value**	**Whole lung**	**(mean ± SD)**	-882.1 ± 68.6	-885.4 ± 35.4	0.87
	**Right lung**	**(mean ± SD)**	-887.1 ± 38.9	-887.3 ± 39.0	0.79
	**Left lung**	**(mean ± SD)**	-881.2 ± 89.1	-883.6 ± 31.4	0.87
**Image noise**	**Whole lung**	**(mean ± SD)**	43.0 ± 17.5	34.9 ± 18.7	< 0.0001
	**Right lung**	**(mean ± SD)**	40.9 ± 14.5	33.5 ± 15.7	< 0.0001
	**Left lung**	**(mean ± SD)**	45.1 ± 19.8	36.4 ± 21.2	< 0.0001
**SNR**	**Whole lung**	**(mean ± SD)**	41.5 ± 6.2	64.6 ± 7.2	< 0.0001
	**Right lung**	**(mean ± SD)**	41.8 ± 5.8	64.8 ± 7.4	< 0.0001
	**Left lung**	**(mean ± SD)**	41.3 ± 6.7	64.4 ± 7.1	< 0.0001
**CPED**	**Whole lung**	**(mean ± SD)**	2.0 ± 1.6	2.8 ± 2.7	0.001
	**Right lung**	**(mean ± SD)**	2.1 ± 1.5	2.0 ± 1.4	0.63
	**Left lung**	**(mean ± SD)**	2.0 ± 1.6	3.6 ± 3.4	0.0001

SNR: signal-to-noise ratio, CPED: cardio-pulmonary edge distance, CPES: cardio-pulmonary edge slope, SD: standard deviation

Corrected version:

**Table 3:** Comparisons of quantitative image quality indices between CT data with and without CLEAR Motion

			CT with CLEAR Motion	CT without CLEAR Motion	p value
CT value	Whole lung	(mean ± SD)	-882.1 ± 68.6	-885.4 ± 35.4	0.87
	Right lung	(mean ± SD)	-887.1 ± 38.9	-887.3 ± 39.0	0.79
	Left lung	(mean ± SD)	-881.2 ± 89.1	-883.6 ± 31.4	0.87
Image noise	Whole lung	(mean ± SD)	43.0 ± 17.5	34.9 ± 18.7	< 0.001
	Right lung	(mean ± SD)	40.9 ± 14.5	33.5 ± 15.7	< 0.001
	Left lung	(mean ± SD)	45.1 ± 19.8	36.4 ± 21.2	< 0.001
SNR	Whole lung	(mean ± SD)	41.5 ± 6.2	64.6 ± 7.2	< 0.001
	Right lung	(mean ± SD)	41.8 ± 5.8	64.8 ± 7.4	< 0.001
	Left lung	(mean ± SD)	41.3 ± 6.7	64.4 ± 7.1	< 0.001
CPED	Whole lung	(mean ± SD)	2.0 ± 1.6	2.8 ± 2.7	0.001
	Right lung	(mean ± SD)	2.1 ± 1.5	2.0 ± 1.4	0.63
	Left lung	(mean ± SD)	2.0 ± 1.6	3.6 ± 3.4	< 0.001
CPES	Whole lung	(mean ± SD)	486.3 ± 233.3	430.1 ± 252.1	0.001
	Right lung	(mean ± SD)	467.8 ± 213.3	497.2 ± 238.0	0.80
	Left lung	(mean ± SD)	504.9 ± 252.3	361.7 ± 249.7	< 0.001

SNR: signal-to-noise ratio, CPED: cardio-pulmonary edge distance, CPES: cardio-pulmonary edge slope, SD: standard deviation.

